# Prospective, comprehensive, and effective viral monitoring in Cuban children undergoing solid organ transplantation

**DOI:** 10.1186/2193-1801-3-247

**Published:** 2014-05-16

**Authors:** Vivian Kourí, Consuelo Correa, Pedro A Martínez, Lizet Sanchez, Alina Alvarez, Grehete González, César E Silverio, Norma Hondal, Jose Florin, Lourdes Pérez, Diana P Duran, Yardelis Perez, Nancy Cazorla, Dalmaris Gonzalez, Juan C Jaime, Alberto Arencibia, Sandra Sarduy, Lissette Pérez, Yudira Soto, Mabel González, Iliana Alvarez, Elvira Dorticós, Juan J Marchena, Luis Solar, Belsy Acosta, Clara Savón, Ulrich Hengge

**Affiliations:** Sexually Transmitted Diseases Laboratory, Virology Department, Institute of Tropical Medicine “Pedro Kourí”, Havana City, Cuba; Epidemiology and Statistic Department, Institute of Tropical Medicine “Pedro Kourí”, Havana City, Cuba; University Pediatric Hospital “William Soler”, Havana City, Cuba; University Pediatric Hospital of “Centro Habana”, Havana City, Cuba; National Institute of Haematology and Immunology, Havana City, Cuba; Respiratory Viruses Laboratory, Virology Department, Institute of Tropical Medicine “Pedro Kourí”, Havana City, Cuba; Haut Zentrum (Skin Center), Duesseldorf, Germany; Virology Department, Institute of Tropical Medicine ¨Pedro Kourí¨, Autopista Novia del Mediodia Km 6., La Lisa, Havana City, Cuba

**Keywords:** Transplant, Pediatric, CMV, Cuba, Viruses

## Abstract

**Purpose:**

In Cuba, viral monitoring in the post-transplant period was not routinely performed. The aim of this research is to identify the most frequent viruses that affect transplanted Cuban children, by implementing a viral follow-up during the post-transplant period.

**Methods:**

The study population included all Cuban pediatric patients who underwent solid organ transplantation (SOT) between November 2009 and December 2012. A total of 34 transplanted pediatric patients of kidney (n = 11) and liver (n = 23) were prospectively monitored during a 34-week period for viral DNAemia and DNAuria by simultaneous detection of cytomegalovirus (CMV), Epstein-Barr virus, herpes simplex virus type 1 and 2, varicella zoster virus, human herpesvirus 6, human adenovirus, and polyomaviruses (BKV and JCV) using quantitative real-time polymerase chain reaction (qRT-PCR).

**Results:**

Viral genome of at least one virus was detected in 21 of 34 recipients, 18 patients excreted virus in urine while 12 presented DNAemia. CMV (41.2%) and BKV (35.3%) were the most frequent viruses detected during the follow-up. CMV was the virus mainly associated with clinical symptoms and DNAemia. Its excretion in urine (with cut off value of 219 copies/mL) was associated with detection in plasma (p < 0.001); furthermore, CMV viruria was predictive of CMV viremia (OR:8.4, CI:2.4-29.1, p = 0.001). There was no association between high viral load and clinical complications, due to the prompt initiation of preemptive ganciclovir. Conclusion: This comprehensive viral monitoring program effectively prevents the development of critical viral disease, thus urge the implementation of qRT-PCR as routine for viral monitoring of transplanted Cuban organ recipients.

## Background

Infections represent one of the most frequent complications among patients undergoing organ transplantation, and among them viral infections constitute a significant cause of morbidity and mortality after solid organ transplantation (SOT). They not only induce specific diseases, but also favor the development of allograft damage, opportunistic infections, and acute rejection (Fishman and Rubin, 1998). Although cytomegalovirus (CMV) is the most common opportunistic pathogen seen in transplant recipients, other viruses may also affect clinical outcome. Among them, other herpesviruses, polyomaviruses and adenoviruses are important (Imperiale and Major, 2007; Rickinson and Kieff, 2007; Roizman et al., 2007; Wold and Horwitz, 2007; Yamanishi et al., 2007).

Major advances in the management of all these viral infections have been achieved because of the availability of novel pharmaceutical agents. In addition, the establishment of polymerase chain reaction (PCR)-based qualitative and quantitative (qRT-PCR) monitoring of viral DNA in blood or serum (DNAemia) has allowed optimal management of antiviral treatment in many countries, as it permits the identification of preclinical or early stages of virus-related pathology (Humar and Michaels, 2006; Martin-Gandul et al., 2013).

However, the appropriate extent of viral monitoring and the critical time window for initiating PCR-guided preemptive antiviral therapy remain controversial. Recent studies on the screening and management of viral infections have focused on CMV, and comprehensive data on viral DNAemia and disease, including CMV and other relevant viruses in the post-transplantation settings, such as Epstein-Barr virus (EBV), herpes simplex virus (HSV), human herpesvirus 6 (HHV6), human adenovirus (ADV), and BK virus (BKV), are sparse. In addition, most previous studies predominantly analyzed adult transplantation cohorts (Schonberger et al., 2010).

The pediatric cohort is at high risk of developing virus-related complications due to immunological immaturity and the increased alloreactivity risk that requires a strong immunosuppressive treatment (Grimaldi et al., 2005).

In 1970, Cuba initiated the transplantation program. Currently, kidney and liver are the more common organ transplants performed in children (Abdo Cuza 2010). However, only serological pre-transplant screening is carried out, whereas specific viral tests (qualitative PCR) are requested when clinical signs and symptoms, suggestive of viral disease, appear. Furthermore, neither quantitative methods for monitoring of viral infections nor preemptive therapy have ever been used in this group of patients. The aim of this research is to identify the most frequent viruses that affect Cuban transplanted children, by implementing an appropriate viral follow-up during the post-transplant period.

## Results

As shown in Table 1, 29 out of the 34 (85.3%) transplanted patients survived after the graft and 25 (73.5%) successfully completed the follow-up, with best results observed for patients with liver transplantation (82.6%, 19/23 patients). Unfortunately, 14.7% of patients died immediately or few days post-transplantation (average 25 days, range 8–48 days), mainly by complications directly linked to the surgery or due to the low Karnofsky performance status at the time of transplantation (Schag et al., 1984; Yates et al., 1980). In addition, 4 patients (11.8%) lost the graft (one with acute rejection (kidney), one with rejection as a result of discontinuing the immunosuppressive therapy (liver), and two with vascular thrombosis (kidney recipients).

**Table 1:** **General information of the transplanted patients studied**

Patients characteristics		Total of patients n = 34 (100%)	Liver n = 23 (100%)	Kidney n = 11 (100%)	
Donor* n(%)	Live (%)	5 (14.7)	4 (17.4)	1 (9.1)	
	Deceased (%)	29 (85.3)	19 (82.6)	10 (90.9)	
Age average average of years (range)		10.4 (1–17)	9.6 (1–17)	13.3 (7–16)	
Cytomegalovirus IgG Pretransplant serology. (n = 32)		29 (90.6)	19 (82.6)	10 (90.9)	
Epstein Barr Virus IgG Pretransplant serology. (n = 32)		29 (90.6)	20 (87.0)	9 (81.8)	
Herpes Simplex Virus IgG Pretransplant serology. (n = 32)		25 (78.1)	17 (73.9)	8 (72.7)	
Immunosuppression regimen (%)		Prednisone	23 (100)	Prednisone	11 (100)
		Cyclosporine (Cs)	13 (56.5)	Cs	7 (63.6)
		or Tacrolimus	11 (47.8)	or Tacrolimus	2 (8.7)
		Mycophenolatemofetil (MMF)	21 (91.3)	If Cs then was indicated MMF	7 (63.6)
		Basiliximab	3(13.0)	Basiliximab	5 (45.5)
				or Thimogam	4 (36.4)
Antiviral prophylaxis (time)		Ganciclovir/valganciclovir (1 month)		Ganciclovir/valganciclovir (3 months)	
		Aciclovir (for two months after Ganciclovir or valganciclovir prophylaxis)			
Graft loss	4 (11.8)	1 (4.3)		3 (27.3)	
Died	5 (14.7)	3 (13.0)		2 (18.2)	
Successful transplant	25 (73.5)	19 (82.6)		6 (54.5)	
Samples analyzed {serum/urine}	578 {289/289}	426** {213/213}		152*** {76/76}	

*Abbreviation*: IgG: Immunoglobulin G.

*All donors were seropositives to CMV, EBV and HSV IgG. **Five patients had an incomplete follow up (only 30 samples). ***Five patients had an incomplete follow up (only 20 samples).

In this cohort of Cuban pediatric recipients, viral DNA was serially monitored for 238 days, on a weekly basis during the first month, then every two weeks until 90 days after transplantation and at monthly intervals, thereafter. Viral DNA was detected in 21 of 34 patients (61.8%) and 22.7% of the screened samples (80/352). The highest positivity was found in urine samples (18 patients, 60/162 samples), compared to plasma (12 patients, 20/190 samples) (Table 2). The median time to first detection of viral DNA following transplantation was 3 weeks (inter quartile range [IQR]: 0–8.5 weeks). In urine, the interval was 4 (IQR: 0–10), while in plasma it was 8 (IQR: 2–12).

**Table 2:** **Distribution of positivity for different viruses, among the patient studied**

Virus	Patients over all*			Liver recipients			Kidney recipients		
	N = 34 (%)			N = 23 (%)			N = 11 (%)		
	Total positive	Plasma	Urine	Total positive	Plasma	Urine	Total positive	Plasma	Urine
Any	21 (61.8)	12 (35.3)	18 (52.9)	15 (65.2)	9 (39.1)	12 (52.2)	6 (54.5)	3 (27.3)	6 (54.5)
CMV	14 (41.2)	10 (29.4)	11 (32.4)	11 (47.8)	7 (30.4)	8 (34.8)	3 (27.3)	3 (27.3)	3 (27.3)
HHV6	1 (2.9)	1 (2.9)	0	1 (4.3)	1 (4.3)	0	0	0	0
EBV	1 (2.9)	1 (2.9)	0	1 (4.3)	1 (4.3)	0	0	0	0
HSV	0	0	0	0	0	0	0	0	0
VZV	0	0	0	0	0	0	0	0	0
BKV	12 (35.3)	2 (5.9)	12 (35.3)	8 (34.8)	0 (0)	8 (34.8)	4 (36.4)	2 (18.2)	4 (36.4)
JCV	4 (11.8)	1 (2.9)	3 (8.8)	2 (8.7)	1 (4.3)	1 (4.3)	2 (18.2)	0	2 (18.2)
ADV	3 (8.8)	1 (2.9)	2 (5.9)	1 (4.3)	1 (4.3)	0	2 (18.2)	0	2 (18.2)

*The total number of patients screened in each fluid does not always coincide with the total of positive patients, since one patient may have been detected with more than one virus.

*Abbreviations*: CMV: Cytomegalovirus, HHV6: Human Herpes Virus 6, EBV: Epstein Barr Virus, HSV: Herpes Simplex Virus, VZV: Varizella Zoster Virus, BKV: BK Virus, JCV: JC Virus, ADV: Adenoviruses.

Liver recipients had detectable viral DNA in 65.2% (15/23 patients) and kidney recipients in 54.5% (6/13 patients). Most patients tested positive for CMV (14/34 patients [41.2%]) or BKV (12/34 patients [35.3%]) (Table 2). In contrast, the other viruses were only rarely detected (HHV6/EBV 1/34 patients each [2.9%]; JC virus (JCV) 4/34 patients [11.8%]; ADV 3/34 patients [8.8%]) and HSV and Varicella Zoster Virus (VZV) were not detected in any patient (Table 2).

Overall, the detection of viruses in plasma was less frequent than in urine and CMV was significantly more frequently detected in this compartment compared to the others viruses (10/12 patients; odds ratio [OR]: 22.5, confidence interval [CI]: 3.5-145.3, p < 0.001) (Table 2). In liver transplant recipients five different viruses were detected in plasma (CMV, HHV6, EBV, JCV and ADV), while in kidney transplanted patients only 2 viruses (CMV and BKV) were diagnosed. No significant association between immunosuppressive regimen and CMV detection was found (p > 0.05).

Six patients were detected with CMV in the two compartments, being more likely that patients having CMV DNA detectable in urine also have CMV DNAemia (OR = 15.1, CI 4.6- 49.9, p < 0.001). Furthermore, CMV excretion in urine was predictive of CMV viremia (OR: 8.4, CI 2.4-29.1, p = 0.001). Despite all donors were seropositive for CMV, EBV and HSV (Table 1), recipient seronegativity was not associated with viral detection (data not shown).

Recent studies have been able to determine the presence of co-infections or mixed infections among viral and non-viral agents. In our study we found that the total of patients with any co-infection (including viral and non-viral) were 8 (23.5%) and 17.6% (6/34) showed viral co-infections either in the same or in different compartments at the same time point (concurrently). CMV-BKV was the most common viral co-infection (3/6), and HHV6-CMV, ADV-BKV, BKV-JCV and EBV-CMV co-infections were also detected. One of these patients had two viral co-infections (CMV-BKV and EBV-CMV) at different time points during the follow up.

### Viral load analysis for the different viruses in each type of fluid

**Figure 1:**
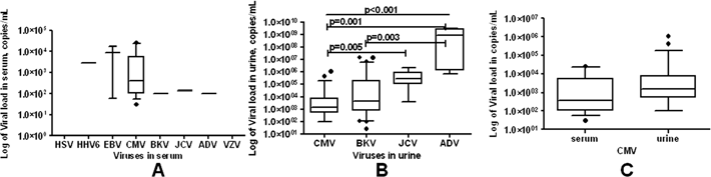
**The figure displays the comparison of the median of viral load among each virus in serum (A), in urine (B), and of CMV between serum and urine (C).** In serum, differences between viral load medians could not be established because there were few viremic patients. In urine, there were statistical differences between the detected viruses. There were also significant higher levels of CMV viral load in urine compared to serum.

Viral load analysis was made for different viruses in each sample type. In urine, the most common viruses detected (CMV and BKV) showed lower viral load (median: 1.5 × 10^3^, ranges 1.0 × 10^2^ to 1.1 × 10^6^ copies/mL and median: 4.7 × 10^3^, ranges 2.7 × 10^1^ to 1.4 × 10^7^ copies/mL, respectively). In contrast, higher viruria load was detected for ADV (median: 8.4 × 10^8^ copies/mL, ranges 6.3 × 10^5^ to 3.0 × 10^9^ copies/mL) and JCV (median: 2.7 × 10^5^copies/mL, ranges 4.1 × 10^3^ to 2.1 × 10^6^ copies/mL); however these viruses were detected less frequently than the others  (Table 2, Figure 1). The viral load levels, among viruses excreted in urine, were significantly different (p < 0.001), particularly CMV and BKV vs. ADV (p = 0.001 and p = 0.003, respectively) and CMV vs. JCV (p = 0.005). Since the detection of other viruses, different from CMV was very rare in plasma, no statistical association could be established.

The median viral loads of CMV between plasma and urine were statistically different (p = 0.03). The highest area under curve (AUC) value, 0.667, was achieved with the CMV viral load in urine, at the cut off 219 copies/mL, it reached a sensitivity of 85.2%, and a specificity of 50% for predicting CMV DNAemia (Figure 1).

### Longitudinal analysis of viral detection in clinical samples from transplanted patients during the follow-up

At the time of transplantation (time 0), mainly those viruses excreted in urine were detected (CMV in two patients, BKV and JCV in three patients each of them). Because of the prophylaxis with ganciclovir (GCV) or valganciclovir (VGV) in months 1–3 after the transplantation, CMV was detected in only one patient. Nevertheless around the weeks 10–14 after transplantation, CMV detection increased to five patients. In addition, BKV excretion was detected throughout the follow-up. The median time to initial CMV, BKV and JCV detection was 11 weeks (IQR: 3.5-12.5), 5 weeks (IQR: 0.75-17.5) and 0 weeks (IQR: 0–0.75) respectively, following transplantation. The time to first detection of each virus was statistically different for JCV vs CMV (p = 0.002) and JCV vs BKV (p = 0.004). EBV, HHV6, and ADV were less frequently found during the longitudinal screening (<10%) and the median time to first detection of those viruses ranged from 2 to 10 weeks after the transplantation. After the week 30, more than 90% of patients under monitoring cleared the viruses from the compartments analyzed.

### Viral detection and clinical impact

CMV was the most frequently detected virus in patients with symptoms and complications during the follow-up. It was found causing direct complications in 3 patients (CMV-syndrome, patients No 22, 23 and 15, Table 3) who were treated with GCV or VGV. This virus was also detected in 4 patients who presented further complications (bacterial, rejection episode, biliary stricture) and were treated with GCV or VGV; 3/4 were already under treatment with antiviral prophylaxis at the moment of the clinical complications (data not shown) and 1/4 received therapeutic treatment (patient No 18, Table 3). CMV was also found in patients without any symptoms or complications (7 patients), 3 of these recipients had viremia and received preemptive antiviral treatment (patients No 1, 8 and 12, Table 3).

**Table 3:** **Characteristics of patients and monitoring of viral load in those receiving preemptive or therapeutic antiviral treatment**

No patient	Type of transplant	Therapy	Weeks of follow-up at beginning therapy (duration in weeks)	Signs and symptoms	Viral load (copies/mL) before therapy	Viral load (copies/mL) after therapy
1	Liver	Valganciclovir	14 (4)	asymptomatic	CMV viremia: 10^3^	No viremia
8	Liver	Valganciclovir	11 (4)	asymptomatic	CMV viremia: 10^4^	No viremia
22	Liver	Valganciclovir	10 (10)	Elevated AST, ALT	Viremia: CMV 10^4^, EBV 10	No viremia
23	Liver	Ganciclovir + Valganciclovir	5 (5 days + 4)	Elevated AST, ALT	Viremia: CMV 10^4^	No viremia
					Urine: CMV 10^3^	Urine: no virus detected
12	Kidney	Valganciclovir	29 (4)	asymptomatic	Viremia: CMV 10, BKV 10	No viremia
					Urine: CMV 10^2^, BKV 10^7^	Urine: CMV 10, BKV 10^4^
15	Kidney	Valganciclovir	22 (4)	Fever	Viremia: CMV 10^2^	No viremia
					Urine: CMV 10^5^	Urine: no virus detected
			34 (12)	Fever and chronic reject	Viremia: CMV 10^2^	No viremia
					Urine: CMV 10^6^	Urine: CMV 10^5^
18	Kidney	Valganciclovir	13 (12)	Urinary infection	Viremia: CMV 10^2^	No viremia
					Urine: CMV 10^3^, BKV 10^2^	Urine: CMV 10^2^

*Abbreviations*: AST: aspartate aminotransferase, ALT: alanine aminotransferase, CMV: Cytomegalovirus, EBV: Epstein-Barr virus, BKV: BK virus.

ADV excretion in urine was associated with a febrile syndrome in one patient, which resolved spontaneously (data not shown).

In total, 7 patients received supplementary preemptive or therapeutic therapy with GCV or VGV, based on the virological results obtained during the follow-up and/or the clinical symptoms (Table 3). All treated patients showed clinical and/or virological evidence of response to the antiviral therapy. No association between viral-related disease and high viral loads was observed, because of the early onset of antiviral therapy in patients with detectable viremia (Table 3).

The median time of GCV or VGV preemptive treatment was 13.5 weeks (IQR: 11.5-25.3 weeks). During preemptive therapy, no toxicities, such as renal or hematologic impairment, were observed.

## Discussion

One of the most important factors for achieving a successful transplantation in children seems to be the status of the patients in the pre-transplant period, since it influences in the early survival after the procedure (Herthelius et al., 2012; Rao et al., 2011). Transplanted patients present a higher susceptibility to the development of infections and surgical complications; these seemed to be the main causes of early death in the current study, because two patients died of vascular thrombosis, another of lung hemorrhage, other with disseminated intravascular coagulation and one case with sepsis. The rate of transplantation success found in the present study is similar to the rates of survival in developing countries (Rao et al., 2011; Wang et al., 2007).

Viral infections remain a significant cause of morbidity and mortality following transplantation. Although CMV is the most common opportunistic pathogen seen in transplant recipients, numerous other viruses have also affected outcome. It has been shown that preventive measures such as pre-transplant screening, prophylactic antiviral therapy, or post-transplant viral monitoring may limit the impact of these infections. Recent advances in laboratory monitoring using quantitative methods and antiviral therapy have further improved outcome.

In Cuba, great efforts have been undertaken in reducing the morbidity and mortality related to the transplant, however, so far no viral monitoring in the post-transplant period was routinely performed and quantitative PCR assays were not available for the screening of transplanted patients. Thus, the present study has allowed, not only assessing the replication of different viruses in samples from pediatric transplanted recipients, but has also contributed to the improvement in the management of their clinical course.

Of note, 61.8% of our patients tested positive for viral DNA at least once, similar to other reports (Schonberger et al., 2010; Verdeguer et al., 2011). In agreement with most of the reviewed literature, CMV represents the most frequent virus detected in transplanted patients (Comoli and Ginevri, 2012). Costa et al., have detected active infection by CMV in 43.3% of liver transplant recipients followed for one year, while HHV6 was detected in 40% (Costa et al., 2011). The present result is also similar to the rate found in a prospective viral monitoring performed in German children undergoing allogeneic hematopoietic stem cell transplantation. They reported an overall detection of any viral DNA in 62.5% of the recipients, with CMV being detected in the serum of 28% of patients while HHV6, ADV and BKV were less frequently detected (Schonberger et al., 2010). Another study performed in Spain, also identified CMV as the most frequent virus (28.9%) among the plasma analyzed (Verdeguer et al., 2011).

The introduction of molecular methods made it possible to detect co-infections with different pathogens in kidney transplant and allogeneic stem cell recipients (Abdo et al., 2009; Yamamoto and Nakamura, 2000; Zawilinska et al., 2008). Co-infections are frequently detected in immunocompromised patients as the altered host immune response is unable to control latent viruses (Bissinger et al., 2005; Pliquett et al. 2012). However, the exact implications of co-infections with regard to the pathogenesis of transplantation-associated diseases are not well known (Sampaio et al., 2012). In the present study, CMV was associated with viral co-infections in blood, which may be predictable, since CMV replication is known to increase the risk of other opportunistic infections (50). In addition, the detection of CMV/BKV co-infection could very likely indicate the presence of significant immunosupression.

It has been demonstrated that the detection of CMV-DNA in plasma by qRT-PCR appears to be an effective prognostic predictor of CMV disease (Martin-Gandul et al., 2013). However, measuring the viral load in specific compartments such as tissues and body fluids may be an alternative way to differentiate between latent infection and reactivation, as well as to assess disease activity in situations where the plasma viral load correlates poorly with disease activity (Kouri et al. 2010; Petrisli et al., 2010). The present finding of the predictive value of CMV viruria with regard to the onset of CMV viremia, together with the fact that a cutoff value of CMV viral load >219 copies/mL in urine is associated with the subsequent detection of CMV in plasma, is an important issue to consider for tracking Cuban transplant patients and indicates the possibility of using quantitative monitoring of CMV in urine, besides plasma, as a diagnostic tool for the early prediction of patients who may develop viremia.

The exact cut-off value of CMV viremia for initiation of preemptive treatment has not yet been defined (dela Torre-Cisneros et al., 2011). In randomized trials in SOT this cut-off has been set at 300000 copies/mL (Gerna et al., 2007). Furthermore, the quantification of virus in urine samples of kidney transplants recipients has proven to be reliable in providing clinically relevant information, particularly for BKV management, where no specific antiviral therapy is currently available and the reduction of the immunosuppressive regimen depends on the viral loads level in urine and plasma specimens (Mischitelli et al., 2008). Thus, in agreement with recent reports (Farfan et al., 2011), the present study reinforces the urgent need of inclusion of qRT-PCR methods for the management of viral infections in the Cuban transplantation program.

The longitudinal analysis of the virus loads monitored during the 34 weeks revealed that, at the time of transplantation (time 0), asymptomatic excretion of several viruses was observed, indicating that the lack of antiviral prophylaxis could jeopardize the success of graft take. Generally, the occurrence of viral DNAemia is determined by the capacity of the immune system to control viral replication. Consistently, the majority of the studied cases with viral DNAemia were detected within the first 100 days post transplantation. CMV replication increased between weeks 10–14 after transplantation, probably due to the discontinuation of antiviral prophylaxis along with the immunosuppression regimen (Razonable, 2013). BKV, although less frequent, was detected in 35.3% of patients and remained detectable during the follow-up. To date, no specific antiviral treatment has proven to be effective, and the only intervention of choice is reduction and/or switching of immunosuppressive drugs (Suwelack et al. 2012).

As expected, CMV was the major cause of morbidity in the present study, although ADV caused disease in one patient. CMV is recognized as the major infectious complication in transplant recipients producing a variety of end-organ diseases or CMV syndrome with fever and leucopenia (Cordero et al., 2012; de la Torre-Cisneros et al., 2011). In addition to directly attributable morbidity, CMV has indirect effects (Caston Osorio and Zurbano Goni 2011), including an immunomodulating activity that is likely responsible for an increased risk of additional opportunistic infections.

One kidney recipient developed an ADV-associated febrile disease, which has been previously described; however, this virus may also produce hemorrhagic cystitis colitis, hepatitis, pneumonitis and encephalitis (Lynch et al., 2011). BKV infection is associated with BKV-associated nephropathy or hemorrhagic cystitis that affects up to 10% of bone marrow- and kidney-transplant patients; however, none of the studied patients developed such a type of complication (Rinaldo and Hirsch, 2007).

Seven patients under specific antiviral therapy (Table 3) were accurately evaluated with regard to the response to the antiviral therapy, confirming that the use of qRT-PCR is critically important for the management of the transplanted patients, as has been described in most of the current international guidelines (Le Page et al., 2013; Tong et al., 2011). Yet, preemptive GCV therapy limited the extent of viral DNAemia and the manifestation of viral disease such that no death from viral DNAemia or disease associated to viral activation occurred in our cohort. We opted for a combination of prophylactic GCV and ACV administration and preemptive GCV therapy.

## Conclusions

We have reported our results on a prospective and comprehensive quantitative PCR-guided viral monitoring program, for simultaneous detection of nine different viruses. Even though the sample size was small, this represented the entire population of Cuban pediatric solid organ transplant recipients during the study period.

Some observed information during the viral monitoring ads new or confirms known information worldwide. However, this is the first study using qRT-PCR for the follow-up of transplanted patients in Cuba.

The results presented herein indicate that many patients develop active viral infections following transplantation. Some patients showed active infection by more than one virus either infected sequentially or concurrently. CMV was the most frequent virus detected in any sample during the follow up and mainly caused clinical symptoms, although the levels of viral load were not correlated with symptoms. This viral monitoring in combination with early initiation of preemptive GCV or VGV should effectively prevent the development of critical viral disease, thus, the implementation of quantitative viral load measurement for routine monitoring could be the best strategy for early clinical intervention and efficient treatment for Cuban patients.

## Methods

### Patient cohort

The study population included all Cuban pediatric patients who underwent SOT between November 2009 and December 2012. A total of 34 consecutive pediatric patients undergoing SOT (23 liver and 11 kidney) at the liver and kidney transplant units for pediatric patients in Cuba (University Pediatric Hospital “William Soler” and University Pediatric Hospital of “Centro Habana”) were studied prospectively for a period of 34 weeks, using qRT-PCR for detection of 9 different viruses. Table 1 provides general information about our cohort.

This research has been approved by the Ethical Committees of the participating institutions, and complies with the principles laid down in the Declaration of Helsinki. Parents provided informed consent with regard to the transplantation procedure, including extended viral monitoring. Clinical and therapeutic data from the transplanted patients were collected through a questionnaire performed by the parents as well as from clinical records of the patients.

Immunosuppressive and prophylactic antiviral therapy was based on the guidelines of the respective study protocols of the Protocolo de Trasplante Hepático en Pediatría, 2005, University Pediatric Hospital “William Soler” and Protocolo de Trasplante renal, 1988, University Pediatric Hospital of “Centro Habana”) (Table 1).

Briefly, immunosuppression for SOT was initiated 4 hours before transplantation and consisted of induction therapy with cyclosporine (Cs) (dosage 8–10 mg/kg/day and adjusted according to the creatinine level and cyclosporinemia level). Mycophenolate mofetil (MMF) was initiated on day 3 at a dosage of 600–1200 mg/m^2^ for 3 months or tacrolimus (dosage 0.1-0.3 mg/kg/day). In addition, 7 kidney and 3 liver recipients were also treated with basiliximab (dosage in ≤30 kg 10 mg and ≥30 kg 20 mg, at day 0 and 4 after the graft) or thimogam (dosage 15 mg/kg/day/14 days). Methylprednisolone (dosage, 10–15 mg/kg) was administered for both types of transplant recipients on the day of transplant that was reduced weekly from 2 mg/kg mg/day until 0.5 mg/kg/day, during the first month following transplantation. At the 2^nd^ and 3^rd^ month 0.25 mg/kg/day were used, and thereafter the dosage was administered every 48 hours.

Recommended levels of cyclosporinemia, for kidney recipients: **C**_**0**_ (1^st^ month: 250–350 ng/mL, 2^nd^ -3^rd^ month: 200–300 ng/mL, 4^th^-12^th^ months: 150–200 ng/mL, after the 1^st^ year: 100–200 ng/mL) and **C**_**2**_ (1^st^ month: 1150–1550 ng/mL, 2^nd^ -3^rd^ month: 800–1200 ng/mL, 4^th^-6^h^ month: 750–900 ng/mL and after the 6^th^ month: up to 650 ng/mL).

Recommended levels of cyclosporinemia, for liver recipients: 1^st^ two weeks: 250–350 ng/mL, 3^rd^ week-3^rd^ month: 200–300 ng/mL, 4^th^-12^th^ months: 150–200 ng/mL, afterwards: 100–150 ng/mL).

Recommended levels of tacrolimus (only used for live donor’s recipients): 0 - 2nd weeks: 10–20 ng/mL, 3rd – 4th weeks: 10 – 15 ng/mL, 2nd – 3rd weeks: 5–15 ng/mL and afterwards: 5–10 ng/mL.

VGC (520 mg/m^2^ every 12 hours) or GCV (5 mg/kg every 12 hours) was administered as prophylaxis for 1 to 3 months for both type of recipients and evaluated according to creatinine levels as well as platelets and leucocytes counts. Additionally, Aciclovir (ACV) was administered for two months following GCV or VGV prophylaxis in liver transplanted patients (Table 1). Preemptive therapy with GCV or VGV (at prophylactic doses) was used for the treatment of CMV infections before the appearance of clinical symptoms and after viremia detection or the increase of viral excretion. The decision of initiation antiviral therapy was made by the attending physician.

Whenever an infectious complication (bacterial, fungal or viral) was detected, the specific treatment was administered according to the guidelines described above. Viral disease was defined according to previously published guidelines (Ljungman et al., 2002).

### Collection of samples

One milliliter of plasma and urine samples was obtained weekly in the first month post-transplant. At thirty to 90 days post-transplantation, samples were collected every two weeks. After 90 days, samples were collected monthly up to 34 weeks (238 days) post-transplant.

### Viral monitoring using the real-time technique

Viral monitoring was performed at the STI Laboratory of the Institute of Tropical Medicine ¨Pedro Kouri¨ (IPK, Havana, Cuba) since it is the only center currently performing molecular diagnosis of viral infections, thus samples from all patients were sent to IPK for testing. Viral detection and quantification of CMV, EBV, HSV type 1 and 2 (HSV1 and HSV2), VZV, HHV6, ADV, and polyomaviruses (BKV and JCV) was prospectively monitored in plasma and urine samples by real-time hybridization assays for 34 weeks after transplantation.

DNA extraction was performed from plasma and urine using QIAmp DNA minikit (Qiagen, Germany) following the recommendations of the manufacturers. Seven different qRT-PCR kits were used for the detection and quantification of CMV, EBV, HSV (including HSV1 and HSV2 subtyping), HHV6, VZV, polyomaviruses (BKV and JCV within the same kit), and ADV, following the protocols and the cycling parameters described by the manufacturers (TIB MOLBIOL, Germany). These kits were designed to be assayed under the LightCycler 1.5 platform (Roche Diagnostics, Germany). They include the mix (Light Mix) of specific primers and probes (hybridization probes) for each specific virus and the viral standards (positive DNA controls) ranging from 10 to 10^6^ copies/μL which allow to construct the standard curve and quantify the viral DNA load in each patient’s sample; in addition the kits also include an internal control.

The qRT-PCR mix for each assay was prepared using Light Mix 2–4 uL (specific for each virus), 2-4uL of LightCycler FastStart DNA Master HybProbe (depending on the specific protocol for each virus) (Roche, Germany), 2.6-7 uL of DNAse/RNAse free water, 1.8-2.4 uL of 25 mM MgCl, and 5uL of the extracted DNA from each sample. The analysis and quantification of the samples were automatically performed by the second derivative maximum method, version 3.3 of the LightCycler software. The results were converted to copies/mL according to the formula (Result in copies/mL = Result in copies/μL × Elution volume in μL/ Sample volume in mL). Clinical samples were considered negative (non detectable viral load) if the crossing point exceeded the cycle 40 or if the viral load was below 10 copies/mL, described as the analytical detection limit of the kits.

### Statistical analysis

For statistical analysis, a database was created using the package SPSS version 17.0. In order to determine an association among clinical features, serological results and/or virological findings, the odds ratio (OR) with 95% of confidence intervals were calculated. The presence of statistically significant association (p), was considered if p ≤ 0.05. Comparisons between different groups of patients or between samples were made by using the Fisher exact test, for categorical data, and for numerical variables the Kruskall-Wallis and Mann–Whitney tests were used.

Quantitative RT-PCR was analyzed in two ways. The detection of any virus in patients/samples was interpreted as qualitative outcome: considering positive all patients or samples with more than 10 copies/mL and negative, below this number. However, with regard to the analysis of the viral load distribution in patients/samples, the results were interpreted as quantitative outcome and the numerical values of the viral load in each clinical sample were considered.

We assessed the discriminative power of the viral load level using recipients-operating characteristic curves (ROC). Their accuracy to discriminate between the viral load of a virus associated with further detection of another virus and/or the assessment of the cutoff viral load in a fluid associated with subsequent excretion of the same virus in the other fluid was classified according to the value of the area under ROC curve (15) in: non-informative (AUC_0.5), less accurate (0.5 < AUC_0.7), moderately accurate (0.7 < AUC_0.9), highly accurate (0.9 < AUC < 1) and perfect (AUC = 1). The value of the viral load with the highest sensitivity, above 50% specificity, for discriminating the detection of other virus was taken as the optimal cut-off point.

## Abbreviations

ACV: Aciclovir
ADV: Human adenovirus
AUC: Area under curve
BKV: BK virus
CI: Confidence interval
CMV: Cytomegalovirus
Cs: Cyclosporine
DNAemia: DNA in blood
EBV: Epstein-Barr virus
GCV: Ganciclovir
HHV6: Human herpesvirus 6
HSV: Herpes simplex virus
IPK: Institute of tropical medicine ¨Pedro Kouri¨
IQR: Inter quartile range
JCV: JC virus
MMF: Mycophenolate mofetil
OR: Odds ratio
PCR: Polymerase chain reaction
ROC: Recipients-operating characteristic curves
SOT: Solid organ transplantation
qRT-PCR: Quantitative real-time polymerase chain reaction
VGV: Valganciclovir
VZV: Varicella zoster virus.
